# Cefoxitin versus carbapenems as definitive treatment for extended-spectrum β-lactamase-producing *Klebsiella pneumoniae* bacteremia in intensive care unit: a propensity-matched retrospective analysis

**DOI:** 10.1186/s13054-023-04712-2

**Published:** 2023-11-01

**Authors:** Tanguy Dequidt, Sylvaine Bastian, Mathieu Nacher, Sébastien Breurec, Michel Carles, Guillaume Thiery, Laurent Camous, Benoit Tressieres, Marc Valette, Jean-David Pommier

**Affiliations:** 1 Infectious Diseases Department, University Hospital of Guadeloupe, Pointe-à-Pitre, France; 2 Laboratory of Clinical Microbiology, University Hospital of Guadeloupe, Pointe-à-Pitre, France; 3 PCCEI, University of Montpellier, INSERM, EFS, University of Antilles, Pointe-à-Pitre, France; 4 Clinical Investigation Center Antilles French Guiana (CIC INSERM 1424), Cayenne Hospital Center, French Guiana, France; 5 Transmission, Reservoir and Diversity of Pathogens Unit, Pasteur Institute of Guadeloupe, Pointe-à-Pitre, France; 6 Faculty of Medicine Hyacinthe Bastaraud, University of Antilles, Pointe-à-Pitre, France; 7 Centre for Clinical Investigation 1424, INSERM, Pointe-à-Pitre/Les Abymes, France; 8 Infectious Diseases Department, University Hospital of Nice, Nice, France; 9 Medical Intensive Care Unit, Saint-Etienne University Hospital, Saint-Priest-en-Jarez, France; 10 Intensive Care Unit, University Hospital of Guadeloupe, Pointe-à-Pitre, France

**Keywords:** *Klebsiella pneumoniae*, Extended-spectrum beta-lactamase (ESBL), Cefoxitin, Carbapenem, Bloodstream infections, Intensive care unit

## Abstract

**Background:**

Despite cefoxitin's in vitro resistance to hydrolysis by extended-spectrum beta-lactamases (ESBL), treatment of ESBL-producing *Klebsiella pneumoniae* (KP) infections with cefoxitin remains controversial. The aim of our study was to compare the clinical efficacy of cefoxitin as definitive antibiotic therapy for patients with ESBL-KP bacteremia in intensive care unit, versus carbapenem therapy.

**Methods:**

This retrospective study included all patients with monomicrobial bacteremia hospitalized in intensive care unit between January 2013 and January 2023 at the University Hospital of Guadeloupe. The primary outcome was the 30-day clinical success defined as a composite endpoint: 30-day survival, absence of relapse and no change of antibiotic therapy. Cox regression including a propensity score (PS) and PS-based matched analysis were performed for endpoint analysis.

**Results:**

A total of 110 patients with bloodstream infections were enrolled. Sixty-three patients (57%) received definitive antibiotic therapy with cefoxitin, while forty-seven (43%) were treated with carbapenems. 30-day clinical success was not significantly different between patients treated with cefoxitin (57%) and carbapenems (53%, *p* = 0.823). PS-adjusted and PS-matched analysis confirmed these findings. Change of definitive antibiotic therapy was more frequent in the cefoxitin group (17% vs. 0%, *p* = 0.002). No significant differences were observed for the other secondary endpoints. The acquisition of carbapenem-resistant *Pseudomonas aeruginosa* was significantly higher in patients receiving carbapenem therapy (5% vs. 23%, *p* = 0.007).

**Conclusions:**

Our results suggest that cefoxitin as definitive antibiotic therapy could be a therapeutic option for some ESBL-KP bacteremia, sparing carbapenems and reducing the selection of carbapenem-resistant *Pseudomonas aeruginosa* strains.

**Supplementary Information:**

The online version contains supplementary material available at 10.1186/s13054-023-04712-2.

## Background

Since the 1990s, the emergence and diffusion of extended-spectrum beta-lactamase-producing Enterobacterales (ESBL-E) has been a global concern [1]. In 2021, the rate of invasive ESBL-E isolates has reached 7.5% in French healthcare settings [2]. ESBL-E infections treatment is becoming challenging due to high frequency of co-resistance mechanisms to other antibiotics classes, such as fluoroquinolones and aminoglycosides; therefore, therapeutic options are often very limited. Carbapenems have long been considered to be the reference treatment for infections caused by ESBL-E [3]. Unfortunately, their large prescription constitutes a strong selective pressure, which has led to the emergence and rapid worldwide spread of carbapenemase-producing Enterobacterales [4].

One possible alternative to carbapenems could be cefoxitin, a cephamycin developed in the 1970s, resistant to ESBL hydrolysis and highly active against ESBL-E in vitro [5, 6]. Currently, cefoxitin is the standard treatment for perioperative surgical prophylaxis, and is proposed as second-line antibiotic therapy for acute ESBL-*Escherichia coli* (ESBL-EC) pyelonephritis in adults [7]. Indeed, cefoxitin and other cephamycins have been shown to be effective in several small cases series of mild infections, such as urinary tract infections and bacteremia without signs of severity [8–11]. In a multicenter retrospective study, Senard et al. showed no significant difference in clinical and microbiological success between cefoxitin and carbapenems as the definitive treatment of ESBL-EC urinary tract infections in men [12].

ESBL-*Klebsiella pneumoniae* (ESBL-KP) infections are also common nosocomial infection, but clinical data on their treatment with cefoxitin are scarce [13–15]. In Guadeloupe University Hospital, the observed incidence rate of nosocomial ESBL-E infections is one of the highest in France, with a large predominance of *Klebsiella pneumoniae* [16, 17]. Since 2015, to limit the use of carbapenems, antibiotic guidelines in our intensive care unit (ICU) proposed cefoxitin rather than carbapenems as definitive treatment in cefoxitin-susceptible ESBL-E systemic infections, whenever clinical condition and microbiological findings are compatible. Until now this therapeutic strategy had not been formally evaluated. The aim of our study was thus to compare the clinical efficacy of cefoxitin as definitive antibiotic therapy for ICU patients with ESBL-KP bacteremia, versus standard carbapenem therapy. Selection of cefoxitin- and carbapenem-resistant Enterobacterales in both groups was also analyzed.

## Methods

### Hospital setting and study design

This retrospective single-center study was conducted between January 2013 and January 2023 at the University Hospital of Guadeloupe (UHG), a 800-bed tertiary-care medical center in Guadeloupe, a Caribbean island forming part of the French West Indies. All consecutive adult patients (age ≥ 18 years) hospitalized in ICU with cefoxitin-susceptible ESBL-KP bloodstream infections were included. Definitive antibiotic therapy was defined as the treatment administered after bacterial identification and antibiotic susceptibility testing, regardless of the initial empirical antibiotic therapy. The choice of definitive antibiotic therapy, either cefoxitin or carbapenem (meropenem or imipenem-cilastatin), was guided by local recommendations, but the final decision was left to the treating physician’s discretion. Exclusion criteria were positive blood cultures with multiple bacteria, treatment by another antibiotic, lack of access to clinical records, patient’s death within 24 h of the onset of bacteremia, definitive monotherapy administered for less than 50% of the total duration of antimicrobial therapy and patient refusal to participate.

### Data collection

Medical charts were retrospectively reviewed for data collection. Baseline characteristics were age, sex, body mass index (BMI), Charlson comorbidity index [18], surgery during the previous 30 days, immunosuppressive therapy, sickle cell disease, radio or chemotherapy in the previous 3 months, underlying disease and time between admission to ICU and bacteremia occurrence. Simplified Acute Physiology Score (SAPS) II [19] was used to assess the patient's severity on admission to the intensive care unit. Illness severity at the time of bacteremia was assessed using Sequential Organ Failure Assessment (SOFA) score [20], Pitt bacteremia score [21], septic shock [22] and mechanical ventilation. Other variables included source of bacteremia, appropriate empirical therapy and duration, time to effective antibiotic therapy, co-infections, other antibiotic therapy administered for another reason and antibiotic posology. Co-infections were defined as any bacterial infections occurring prior to ESBL-KP bacteremia and still being treated. Empirical antibiotic therapy was considered appropriate if treatment regimens demonstrated in vitro activity against ESBL-KP and if the first dose was administered within the first 24 h after the blood culture. Time to effective antibiotic therapy was defined as the time elapsed between blood culture collection and start of active antibiotic therapy against the bacteria. *K. pneumoniae* isolates were considered as community-acquired if obtained within 48 h of admission, and hospital-acquired beyond that time. Follow-up was carried out up to 30 days after inclusion.

### Primary and secondary endpoints

The primary endpoint was the 30-day clinical success rate defined as a composite endpoint: 30-day survival after inclusion, absence of relapse and no change of antibiotic therapy before the planned end of treatment.

Secondary endpoints were (i) all-cause mortality at 7 and 30 days post-inclusion, (ii) relapse of infection, defined as a new ESBL-KP bacteremia between the end of treatment and 30 days, (iii) change of antibiotic therapy before the scheduled end of treatment due to the onset of a new co-infection, or caused by clinical or microbiological failure, (iv) microbiological failure defined as the persistence of ESBL-KP-positive blood culture after two days of definitive antibiotic therapy until the end of treatment and (v) selection of all bacteria resistant to cefoxitin or carbapenems identified in any microbiological sample after 24 h of definitive antibiotic therapy until the end of follow-up.

### Microbiological analysis and susceptibility testing

All clinical isolates were identified by standard methods, using Api 20E system and matrix-assisted laser desorption ionization-time of flight mass spectrometry (MALDI-TOF MS, VITEK® MS system, bioMérieux, Marcy l’Etoile, France), according to the manufacturer’s recommendations. Susceptibility to antibiotics was determined by the disk diffusion method on Mueller–Hinton agar (bioMérieux, Marcy l’Étoile, France) according to the guidelines of the Antibiogram Committee of the French Society of Microbiology—European Committee on Antimicrobial Susceptibility testing (CA-SFM-EUCAST). The minimum inhibitory concentrations (MICs) for cefoxitin were measured by diffusion method with MICs test strips (Liofilchem, Roseto degli Abruzzi, Italy). *Klebsiella pneumoniae* strains that showed resistance to third-generation cephalosporins were tested for the production of ESBL by the double-disk synergy and the combination disk methods on agar media, according to the guidelines of CA-SFM-EUCAST. Since 2020, the CA-SFM-EUCAST classified isolates as “susceptible with standard dosing regimen,” “susceptible, increased exposure” or “resistant” to antibiotics [23]. Susceptible strains were defined by minimum inhibitory concentration (MIC) ≤ 8 mg/L for cefoxitin and MIC ≤ 2 mg/L for carbapenems; resistant strains by MIC > 16 mg/L for cefoxitin, MIC > 4 mg/L for imipenem and MIC > 8 mg/L for meropenem; “susceptible, increased exposure” strains correspond to MIC values between the previous two.

### Statistical analysis

Quantitative variables were expressed as mean ± standard deviation (SD) and qualitative variables were expressed as numbers and percentages. Characteristics of the cefoxitin and carbapenem groups were compared using Student’s *t* tests or Mann–Whitney for continuous variables and Chi-square tests or Fisher’s exact tests when appropriate for categorical variables. Kaplan–Meier curves and nonparametric (log-rank) test were used to compare survival in each group.

To identify factors associated with clinical failure, univariate and multivariate analysis using Cox proportional hazards regression were performed. All variables with a *P* value ≤ 0.2 in the univariate analysis were included in a stepwise procedure, and the final multivariate model was obtained when the Akaike information criterion reached its minimum.

In order to reduce selection bias in our two non-randomized treatment groups and explore the causal effect of treatment, we estimated propensity scores (PS) through logistic regression as the logistic transform of the probability of receiving cefoxitin. We selected the variables differing between the two groups, or considered as potential mortality risk factors in previous studies or in our own. In addition to P values, we indicated the standardized mean differences (SMD) to measure covariate balance. As Cohen described, SMD values < 0.2 meant significant balance between groups, values of 0.2–0.5 were considered small differences, values of 0.5–0.8 as medium and values > 0.8 as large [24]. To perform PS-adjusted analysis, a model including PS and definitive antibiotic therapy was built, and then, hazard ratios (HRs) and 95% confidence intervals (CI) were calculated. The PS was also used for matching. Patients treated with cefoxitin of carbapenem were matched (1 to 1) without replacement using the nearest neighbor matching algorithm.

All tests were 2-tailed and *P* values ≤ 0.05 were used for statistical significance testing. Data were analyzed using STATA® software (version 18, StataCorp, College Station, Texas).

### Ethics

All the procedures were in accordance with the 1964 Declaration of Helsinki and its later amendments. Participants received an information notice mentioning their participation to the study and giving the possibility of objecting, according to French legislation. The study was approved by Ethical Committee of the UHG (N°A105_24/04/2023).

### Results

#### Baseline characteristics of patients

**Fig. 1:**
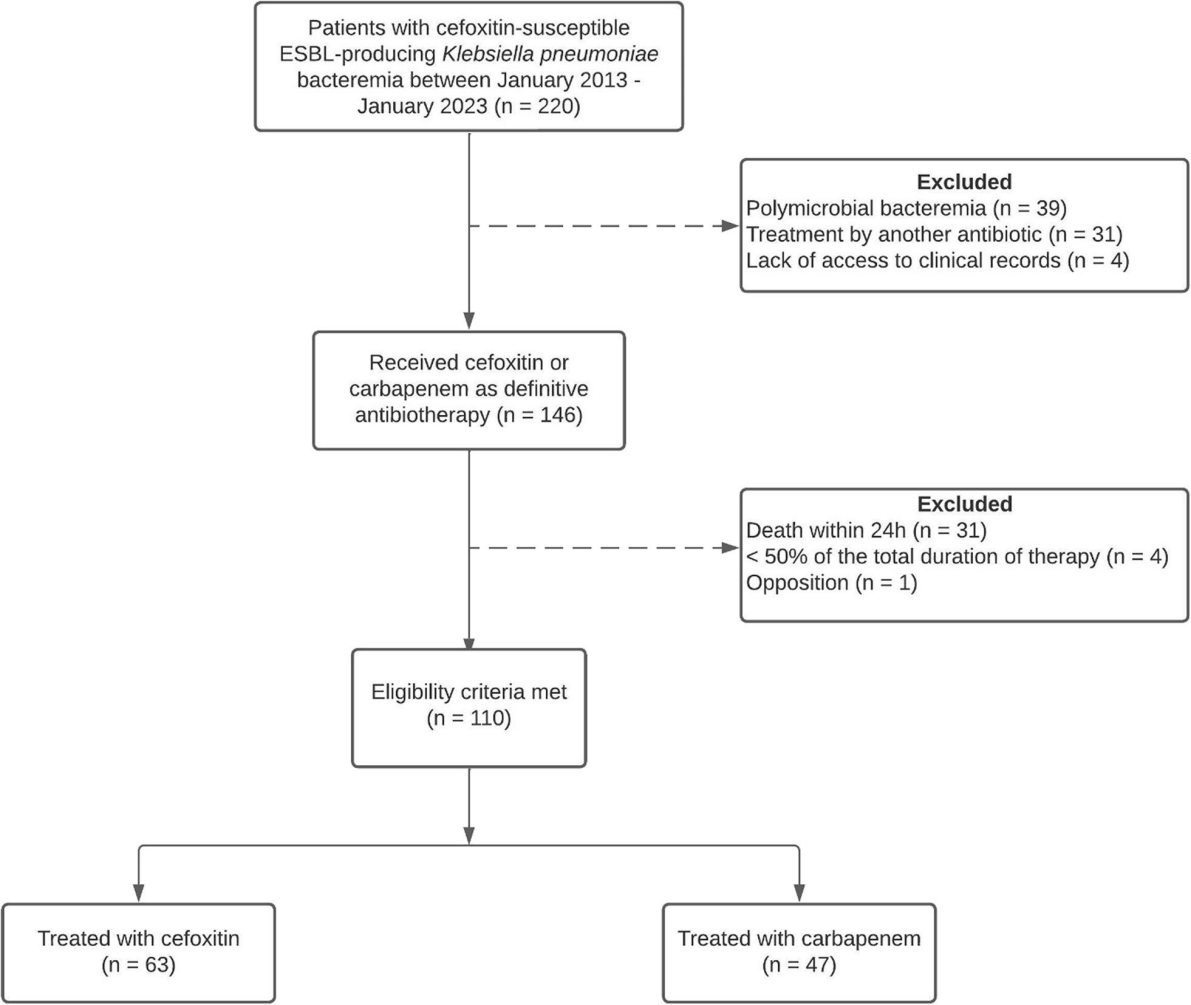
Study flow chart

Over a ten-year period, a total of 220 patients with ESBL-producing *Klebsiella pneumoniae* bacteremia were examined, and 110 patients (50%) were included in the final analysis (Fig. 1). Sixty-three patients (57%) received definitive antibiotic therapy with cefoxitin, while forty-seven (43%) were treated with carbapenems. In the carbapenem group, 22 patients (47%) were treated before 2015 and twenty-five (53%) after 2015. In the cefoxitin group, all patients but one were treated after 2015. Baseline characteristics of patients are summarized in Table 1. Notable differences in clinical characteristic existed between the two treatment groups. Patients who received carbapenems as definitive antibiotic therapy had a higher SOFA score at the time of bacteremia (11 ± 5 vs 9 ± 4, *p* = 0.046) and more often required mechanical ventilation (87% vs 63%, respectively *p* = 0.005) than patients who received cefoxitin. Pneumonia and neuromeningeal infections as a source of bacteremia were statistically more frequent in the carbapenem group (30% vs 11% *p* = 0.014, 8% vs 0% *p* = 0.031, respectively), while urinary tract infections tended to be more frequent in patients treated by cefoxitin (6% vs 21%, *p* = 0.054). Cefoxitin administration was mostly discontinuous, with dosing of 6 g per day (*n* = 30/55, 58%), 8 g per day (*n* = 17/55, 32%) or adapted to renal function (*n* = 5/55, 10%). Three patients (6%) received continuous administration of cefoxitin. In the carbapenem group, 35 patients (74%) were treated with the combination of imipenem and cilastatin, while 12 patients (26%) received meropenem.

**Table 1:** Characteristics in baseline population and PS-matched patients with bacteremia caused by ESBL-producing *Klebsiella pneumoniae* treated either with cefoxitin or carbapenem

Characteristics	Baseline population				PS-matched patients			
	Cefoxitin *n* = 63 (57%)	Carbapenem *n* = 47 (43%)	SMD	*p* value	Cefoxitin *n* = 42 (50%)	Carbapenem *n* = 42 (50%)	SMD	*p* value
Age, *years*	57 ± 17	57 ± 13	< 0.001	.585	55 ± 17	57 ± 12	.121	.864
≥ 65	27 (42)	18 (40)	.093	.630	17 (40)	15 (36)	.098	.653
Male	42 (67)	31 (66)	.015	.938	29 (69)	26 (62)	.151	.491
Year of inclusion								
2018–2022	32 (51)	25 (53)	.048	.803	23 (55)	22 (52)	.048	.827
Body-mass index	27 ± 5	27 ± 8	.123	.582	27 ± 5	28 ± 8	.140	.567
Charlson comorbidity index	4 (2–6)	3 (1–4)	.328	.141	3 ± 3	3 ± 2	.161	.664
Surgery, previous 30 days	21 (34)	13 (28)	.121	.535	14 (33)	11 (26)	.142	.518
Immunosuppressive therapy	4 (6)	6 (13)	.224	.319	4 (9)	6 (14)	.157	.520
Sickle cell disease	2 (3)	2 (4)	.059	1.00	1 (2)	2 (5)	.134	.616
Radio or chemotherapy within the last 3 months	1 (2)	1 (2)	.041	1.00	1 (2)	1 (2)	.004	1.00
Unknown disease	0 (0)	1 (2)	.211	.426	0 (0)	1 (2)	.224	.494
Origin			.021	.913			.101	.645
Community-acquired (ref)	5 (8)	4 (9)			2 (5)	3 (7)		
Nosocomial infection	58 (92)	43 (91)			40 (95)	39 (93)		
Source of bacteremia:								
Central line associated	24 (38)	12 (26)	.272	.165	17 (41)	11 (26)	.306	.165
With thrombophlebitis	9 (15)	3 (7)	.262	.188	5 (12)	3 (7)	.163	.713
Pneumonia	7 (11)	14 (30)	.476	**.014**	7 (17)	12 (29)	.287	.192
Urinary tract	13 (21)	3 (6)	.426	.054	4 (10)	3 (7)	.086	1.00
Intra-abdominal	8 (13)	4 (9)	.136	.489	6 (14)	4 (10)	.147	.738
Liver abscess	1 (1)	1 (2)	.040	1.00	1 (2)	1 (2)	< 0.001	1.00
Skin and soft tissue	2 (3)	0 (0)	.256	.506	1 (2)	0 (0)	.221	1.00
Neuromeningeal	0 (0)	4 (8)	.431	**.031**	0 (0)	3 (7)	.392	.241
Unknown	8 (13)	9 (19)	.177	.428	6 (14)	8 (19)	.128	.558
Time between admission to ICU and bacteremia, *days*	12 ± 13	15 ± 13	.252	.053	12 ± 11	14 ± 12	.166	.485
SAPS II admission score	47 ± 17	49 ± 22]	.111	.557	48 ± 17	51 ± 21	.168	.443
SOFA score at inclusion	9 ± 4]	11 ± 5	.415	**.046**	10 ± 4	11 ± 5	.297	.193
Pitt bacteremia score at inclusion	6 ± 3	7 ± 3	.273	.062	7 ± 3	7 ± 3 (6–9)	.129	.303
Septic shock	25 (40)	19 (40)	.015	.937	17 (40)	18 (43)	.048	.825
Mechanical ventilation	40 (63)	41 (87)	.573	**.005**	33 (78)	37 (88)	.258	.380
Appropriate empirical therapy	31 (56)	28 (65)	.180	.380	22 (52)	27 (64)	.251	.260
Duration of empirical therapy, *days*	1 ± 1	1 ± 2	.113	.788	1 ± 1	1 ± 2	.110	.765
Time to effective antibiotic therapy, *hours*	26 ± 25	12 ± 35	.460	.075	26 ± 24	11 ± 36	.493	.065
Co-infection at inclusion	11 (18)	4 (9)	.234	.208	8 (19)	6 (14)	.251	.591
Other antibiotic therapy at inclusion	12 (19)	5 (11)	.208	.298	8 (19)	7 (17)	.120	.816

Data are presented as mean ± SD or count (%). *p* values in bold are statistically significant. SMD = standardized mean difference,

*ICU* = intensive care unit, *SAPS II* simplified acute physiology score II, *SOFA* sequential organ failure assessment score

### Primary outcomes

The 30-day clinical success was not significantly different for the cefoxitin group (57%) compared with the carbapenem group (53%, *p* = 0.823) (Table 2). Multivariate analysis using Cox regression showed that SOFA score (HR 1.1 [95% CI 1.1–1.2], *p* < 0.001), pneumonia (HR 3.3 [95% CI 1.7–6.4], *p* = 0.001) and intra-abdominal infections (HR 2.4 [1.0–5.6] *p* = 0.044) were independent risk factors of clinical failure (Additional file 1: Table S1).

**Table 2:** Outcomes of patients with ESBL-KP bacteremia according to antibiotic treatment

Outcomes	Cefoxitin (*n* = 63)	Carbapenem (*n* = 47)	Univariate analysis		PS-adjusted analysis (*n* = 100)	
			HR (95%CI)	*p* value	aHR (95%CI)	*p* value
30-day clinical success	36 (57)	25 (53)	0.9 (0.5–1.6)	.823	1.3 (0.6–2.5)	.497
30-day all-cause mortality	18 (29)	20 (42)	0.6 (0.3–1.2)	.131	0.8 (0.4–1.7)	.549
Relapse	6 (11)	4 (11)	1.1 (0.3–3.9)	.887	0.8 (0.1–4.0)	.768
Change of antibiotic	11 (17)	0 (0)	N/A	**.002**	N/A	N/A
7-day all-cause mortality	9 (14)	7 (15)	0.9 (0.3–2.5)	.889	1.5 (0.5–4.8)	.463
Microbiological failure	10 (16)	7 (15)	1.1 (0.4–2.8)	.897	1.6 (0.5–5.3)	.414

Data are presented as median [IQR] or count (%). *p* values in bold are statistically significant. aHR = adjusted hazard ratio. N/A = not applicable

*Fisher's exact test used for this variable for which one of the counts is 0

### Secondary outcomes

**Fig. 2:**
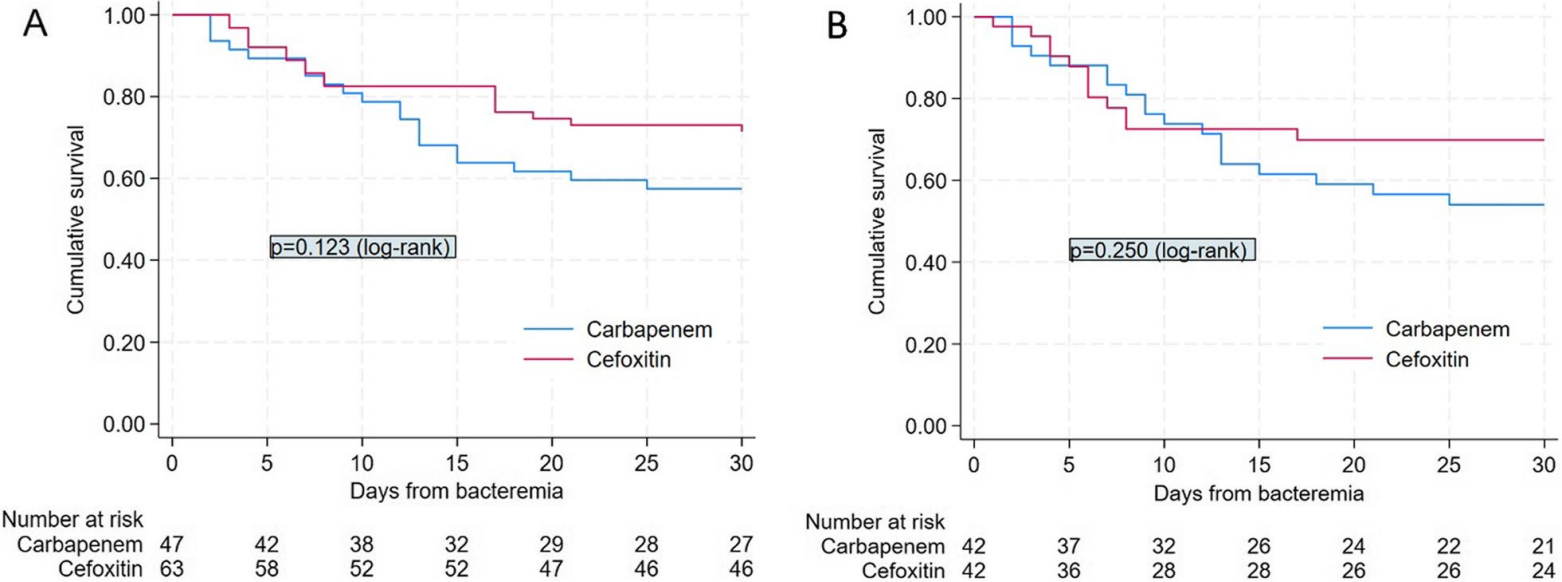
Kaplan–Meier survival curves for baseline population (**A**) and PS-matched patients (**B**) receiving cefoxitin or carbapenem therapy for extended-spectrum beta-lactamase (ESBL)-producing *Klebsiella pneumoniae*

For 30-day survival, Kaplan–Meier curves are illustrated in Fig. 2A. Seven-day all-cause mortality, relapse rates and microbiological failure rates were not significantly different between the two groups in univariate analysis (Table 2). No change of treatment was done in patients treated with carbapenems, while 11 patients treated with cefoxitin (17%) benefited from a change of treatment (*p* = 0.002). The onset of co-infection with cefoxitin-resistant bacteria (one peritonitis and two bloodstream infections caused by *Enterobacter cloacae*, one bacteremia caused by *Pseudomonas aeruginosa*) was the reason for change in 4 patients out of 11 patients (36%), while clinical and microbiological failure were the cause for change in 2 (18%) and 5 patients (46%), respectively. The duration of cefoxitin antibiotic therapy before switching was 5 ± 2 days. Clinical success was, respectively, achieved by 16 (53%), 8 (47%) and 5 (100%) patients treated with cefoxitin at a discontinuous posology of 6 g/day (*n* = 30) to 8 g/day (*n* = 17) and adapted to renal function (*n* = 5), with no significant difference (*p* = 0.10).

### Propensity score analysis for primary and secondary endpoints

The PS score was computed using the covariates age, Charlson comorbidity index, year of inclusion, SAPS II score, source of infection, time to effective antibiotic therapy, mechanical ventilation, septic shock, SOFA and Pitt bacteremia scores. PS-adjusted analysis confirmed that there was no significant difference between the cefoxitin and carbapenem groups for primary and secondary endpoints (Table 2). Regarding the PS-based matched analysis, all variables were well balanced into the five blocks. Forty-two pairs of patients treated with cefoxitin or carbapenem were matched according to PS (Table 1). To assess the quality of matching, we compared the plot of the distribution (Additional file 1: Figure S1). No significant differences were found between the two matched groups for any of the covariates considering P values. All variables had a SMD < 0.5. PS-based matched analysis maintained that there was no significant difference between the cefoxitin and carbapenem groups on 30-day clinical success (HR 0.9 [95% CI 0.5–1.6], *p* = 0.681), 30- and 7-day all-cause mortality (HR 0.6 [95% CI 0.3–1.2], *p* = 0.118 and HR 0.8 [95% CI 0.3–2.5], *p* = 0.739, respectively), relapse (HR 1.0 [95% CI 0.2–4.8], *p* = 0.968) and microbiological failure rates (HR 0.9 [95% CI 0.3–2.5], *p* = 0.782)**.** Kaplan–Meier curves on matched patients showed no significant difference for 30-day survival (*p* = 0.250) (Fig. 2B).

### Subgroup analysis by source of infection

Subgroup analysis of 30-day clinical success showed no significant difference between the cefoxitin and carbapenem groups according to source of infection (Table 3). Failure rates were high when the source of infection was pneumonia or intra-abdominal infection (81% and 75%, respectively), and lower when the source of infection was a central line or urinary tract infection (17% and 37%, respectively). Of the four clinical failures associated with catheter infection in patients treated with cefoxitin, three were related to thrombophlebitis, while the fourth did not undergo Doppler ultrasound evaluation. Among cefoxitin-treated patients who switched treatment, the source of infection was catheter-related for two, pneumonia for two, urinary for one, intra-abdominal infection for one and liver abscess for one.

**Table 3:** Subgroup analysis of 30-day clinical success according to source of infection

30-day clinical success	Cefoxitin *n* = 63 (57%)	Carbapenem *n* = 47 (43%)	OR (95% CI)	*p* value
Source of bacteremia				
Central line associated	20/24 (83%)	10/12 (83%)	1.0 (0.1–8.5)	1.00
Pneumonia	1/7 (14%)	3/14 (21%)	0.6 (0.1–10.0)	1.00
Urinary tract	8/13 (61%)	2/3 (67%)	0.8 (0.1–19.7)	1.00
Intra-abdominal	2/8 (25%)	1/4 (25%)	1.0 (0.1–78.4)	1.00
Unknown	4/8 (50%)	7/9 (78%)	0.3 (0.2–3.4)	.335

Data are presented as count (%). OR = odd ratio

### Microbiology and selection of resistant bacterial strains

All isolates were susceptible to cefoxitin with a median MIC of 4 mg/L (IQR 3–4), and to carbapenems. There was no significant difference in the selection of cefoxitin-resistant bacteria, including *Klebsiella pneumoniae*, between the two groups (Table 4). Fifteen beta-lactamase AmpC-producing Enterobacterales (12 *Enterobacter cloacae* and 3 *Enterobacter aerogenes*) were selected among the cefoxitin-treated patients, compared with only two (1 *Enterobacter cloacae* and 1 *Serratia marcescens*) in the carbapenem group (p = 0.006). In patients receiving carbapenem therapy, the acquisition of *Pseudomonas aeruginosa* (colonization or infection) and among them carbapenem-resistant *Pseudomonas aeruginosa* were significantly higher (respectively, 38% vs 21% *p* = 0.042, 23% vs 5% *p* = 0.007). Among the six carbapenem-resistant *Pseudomonas aeruginosa* infections in the carbapenem-treated group, five appeared after treatment of ESBL-KP bacteremia had been completed, while one occurred during treatment. In the latter case, carbapenem was maintained and colistin therapy was added. Only one of the carbapenem-resistant *Pseudomonas aeruginosa* strains was multidrug-resistant.

**Table 4:** Selection of cefoxitin-resistant bacterial strains at 30 days on any sample after administration of cefoxitin or carbapenem

Characteristic	Cefoxitin *n* = 63	Carbapenem *n* = 47	*p* value
Selection of at least one cefoxitin-resistant gram-negative bacteria at 30 days	30 (48)	22 (47)	.933
*Klebsiella pneumoniae* cefoxitin-resistant	7 (11)	4 (8)	.755
Including colonization	4 (6)	1 (2)	
Including infection	3 (5)	3 (6)	
AmpC β-lactamase-producing Enterobacterales	15 (24)	2 (4)	**.006**
Including colonization	6 (9)	0 (0)	
Including infection	9 (14)	2 (4)	
*Pseudomonas aeruginosa*	13 (21)	18 (38)	**.042**
Including colonization	3 (5)	8 (17)	
Including infection	10 (16)	10 (21)	
*Pseudomonas aeruginosa* carbapenem-resistant	3 (5)	11 (23)	**.007**
Including colonization	2 (3)	5 (11)	
Including infection	1 (2)	6 (13)	
*Stenotrophomonas maltophilia*	0 (0)	1 (2)	.427
*Acinetobacter baumannii*	2 (3)	0 (0)	1.00

Date are presented as count (%). *P* values in bold are statistically significant

## Discussion

To the best of our knowledge, this original study is the first to compare the efficacy of cefoxitin versus carbapenems as definitive treatment in ESBL-*Klebsiella pneumoniae* bacteremia in ICU. The study’s main finding, confirmed by PS-matched analysis, shows that there was no statistical difference in 30-day clinical success when cefoxitin was used as definitive antibiotic therapy for ESBL-KP bacteremia, compared with carbapenems. The second interesting finding is that the use of cefoxitin instead of carbapenems reduced the selection of carbapenem-resistant *Pseudomonas aeruginosa* in these critically ill patients, often at risk of new nosocomial infection.

In our work, cefoxitin appears to be effective in severe infections compared with carbapenems. As expected in ICU, the overall clinical success rate was low (57% in the cefoxitin group), but not lower than in the carbapenems group (53%) and slightly higher than results recently published in a study of ESBL-E infections (including 25 *Klebsiella pneumoniae* isolates) treated with cefoxitin in critical care which reported a therapeutic success rate of 37% [25]. Although patient severity appeared similar in both studies, our recurrence rate seemed lower (11% vs. 27%) as was the rate of antibiotic change (17% vs. 32%). This may be due to the higher number of pneumonias in their study, which was an independent risk factor for clinical failure in ours. However, 30-day mortality does not seem to be different in both studies (29%). Similar to our findings, the mortality rate between patients treated with flomoxef or cefmetazole, another cephamycins and those treated with carbapenem for ESBL-E bacteremia was not significantly different in previous studies [10, 13]. In contrast to our results, two studies comparing flomoxef with carbapenems showed excess mortality in patients treated with cephamycin [26, 27]. The first, conducted in a hemodialysis unit, focused on ESBL-KP bacteremia related to fistula, graft or catheter infections [26], while the second involved a large sample (*n* = 389) of ESBL-EC and ESBL-KP bloodstream infections of various origins and from any hospital ward [27]. The Infectious Diseases Society of America (IDSA) thus does not suggest the use of cephamycins for the treatment of ESBL-E infections until more clinical outcomes data are available [28].

In our study, unlike cefoxitin, no change of treatment was made in the carbapenem group. First of all, carbapenems are the reference, broad-spectrum, last-line treatment. In the absence of rapid clinical improvement in a severe patient or when the source of infection is not controlled, cefoxitin might be changed early as it is not the reference treatment, while carbapenem should only be changed in the event of proven microbiological failure. Another reason is that cefoxitin was systematically switched when co-infections with cefoxitin-resistant bacteria occurred, whereas carbapenem was maintained when there was co-infection with carbapenem-resistant bacteria, and another antibiotic was added.

Many studies of bloodstream infections have shown that the source of infection is a major prognostic factor in clinical success and mortality [29–31]. Interestingly, 100% of patients with catheter-related bacteremia for whom thrombophlebitis was ruled out (*n* = 20) were cured on cefoxitin. Given the total absence of studies on this specific population in the literature, it would be interesting to carry out further studies to confirm these promising results. In contrast, it is difficult to conclude on the efficacy of cefoxitin in bacteremia secondary to pneumonia and intra-abdominal infections, in view of the extreme severity of our patients in the study.

Cefoxitin as definitive treatment for ESBL-KP bacteremia remains controversial. Indeed, this is explained by the description of clinical failures caused by the in vivo acquisition of cefoxitin-resistant mutant *K. pneumoniae* [15]. In 1989, the first treatment failure case of nosocomial pneumonia with cefoxitin (after initial treatment with imipenem-cilastatin) due to the selection of cefoxitin-resistant *K. pneumoniae* was reported [14]. Since then, the mechanism of resistance has been identified as the loss of an outer membrane porin called OmpK36, which provides a channel that allows a wide range of antibiotics to penetrate the *Klebsiella pneumoniae* cell wall [32, 33]. In our study, there was no greater selection of cefoxitin-resistant *K. pneumoniae* in patients treated with cefoxitin than with carbapenems within 30 days following the beginning of the treatment. Interestingly, a slower growth of the bacteria was observed when they lost the OmpK36 porin probably due to the fitness cost, a decrease in the virulence of the strain and an increased susceptibility to neutrophil phagocytosis [34].

Although our study shows no difference in the total number of cefoxitin-resistant bacteria selected between the two groups, some results are interesting. Firstly, as expected, there were more beta-lactamase AmpC-producing Enterobacterales selected in patients previously treated with cefoxitin. Secondly, carbapenem-treated patients selected significantly more *Pseudomonas aeruginosa*, including carbapenem-resistant strains, within 30 days of bacteremia. Cefoxitin therefore might be interesting to avoid the administration of carbapenems, in line with the current policy of carbapenem sparing.

There are some limitations with our study. First, this was a non-randomized retrospective observational study with the inherent shortcomings of these studies. At baseline, our two treatment groups were not comparable on several criteria, with more severe patients overall in the carbapenem group, probably due to an indication bias. Nevertheless, the variables with potential impact on outcome were searched and a PS-based matched analysis was performed to control for potential bias on antibiotic selection. It should be emphasized that considering SMD values, some differences existed between the two groups after PS matching but with a SMD value < 0.5. Second, although our study contains, to our knowledge, the largest published number of patients with ESBL-KP bacteremia treated with cefoxitin in ICU, the sample size was still small. Third, although there were no significant differences in primary and secondary endpoints, using a PS-based matched analysis, we acknowledge that the 95% confidence intervals for propensity-matched outcomes are very wide and include the possibility of harm. Fourth, cefoxitin was mainly administered on a discontinuous basis, whereas several recent studies confirm that continuous administration of large doses of cefoxitin appeared necessary to achieve the recommended beta-lactam PK/PD target in critically ill patients [6, 25, 35].

In conclusion, our results suggest that cefoxitin as definitive antibiotic therapy could be an alternative for ESBL-KP bacteremia in intensive care unit, especially if its origin is a central catheter or urinary tract infection, allowing carbapenem sparing and less selection of carbapenem-resistant *Pseudomonas aeruginosa* strains. Further research, such as prospective interventional studies, is needed to define the exact efficacy of cefoxitin and confirm our findings.

### Supplementary Information

**Additional file 1**. **Table S1:** Analysis of risk factors for 30-day clinical failure in patients with ESBL-KP bacteremia. **Table S2:** Matching graph of the propensity score.

## Data Availability

The data presented in this study will be available from the corresponding author on reasonable request and provided all regulatory and privacy requirements are fulfilled.
