# Supplementary material for: Cefoxitin versus carbapenems as definitive treatment for extended-spectrum β-lactamase-producing Klebsiella pneumoniae bacteremia in intensive care unit: a propensity-matched retrospective analysis
Source: Crit Care. 2023 Nov 1;27:418. doi: 10.1186/s13054-023-04712-2 (PMC10619259; doi:10.1186/s13054-023-04712-2)
Supplement: Supplementary file 1 — Additional file 1. Table S1: Analysis of risk factors for 30-day clinical failure in patients with ESBL-KP bacteremia. Table S2: Matching graph of the propensity score. [file 13054_2023_4712_MOESM1_ESM.docx]

**Supplementary Table 1 – Analysis of risk factors for 30-day clinical failure in patients with ESBL-KP bacteremia**

| **Characteristics** | **30-day success**  n = 61 | **30-day failure**  n = 49 | **Univariate analysis** | | **Multivariate analysis** | |
| --- | --- | --- | --- | --- | --- | --- |
|  |  |  | **HR (95%CI)** | ***p* value** | **aHR (95%CI)** | ***p* value** |
| Age, *year* | 57 [45-67] | 64 [55-69] | 1.0 (1.0-1.0) | .102 | 1.0 (1.0-1.0) | .134 |
| Male | 40 (66) | 33 (67) | 1.0 (0.6-1.9) | .938 |  | |
| Year of inclusion  2018 – 2022 | 34 (56) | 23 (47) | 0.8 (0.4-1.4) | .409 |  | |
| Body-mass index | 25 [22-29] | 26 [24-30] | 1.0 (1.0-1.1) | .780 |  | |
| Charlson comorbidity index | 3 (1-5) | 4 (2-5) | 1.1 (1.0-1.2) | .263 |  | |
| Sickle cell disease | 1 (2) | 3 (6) | 1.7 (0.5-5.4) | .385 |  | |
| Surgery, previous 30 days | 16 (27) | 18 (37) | 1.4 (0.8-2.4) | .301 |  | |
| Type of surgery:  Cardiovascular  Thoraco-abdomino-pelvic  Orthopedic  Neurosurgery  Plastic | 1 (6)  7 (44)  4 (25)  3 (19)  1 (6) | 1 (5)  9 (50)  3 (17)  3 (17)  2 (11) | 1.3 (0.2-10.1)  1.3 (0.5-3.3)  0.7 (0.2-2.6)  0.8 (0.2-2.6)  1.0 (0.2-4.6) | .778  .554  .636  .664  .944 |  | |
| Immunosuppressive therapy | 5 (8) | 5 (10) | 1.2 (0.5-3.0) | .731 |  | |
| Origin:  Community-acquired  Nosocomial infection | 6 (10)  55 (90) | 3 (6)  46 (94) | Reference variable  1.3 (0.4-4.3) | .625 |  | |
| Source of bacteremia:  Central line associated  With thrombophlebitis  Pneumonia  Urinary tract  Intra abdominal  Others*  Unknown | 30 (49)  8 (13)  4 (7)  10 (16)  3 (5)  3 (5)  11 (18) | 6 (12)  4 (8)  17 (35)  6 (12)  9 (19)  5 (10)  6 (12) | 0.2 (0.1-0.5**)**  0.7 (0.2-1.9)  3.5 (1.9-6.4)  0.7 (0.3-1.6)  2.3 (1.1-4.8)  1.8 (0.7-4.5)  0.7 (0.3-1.6) | **.001**  .473  **<.001**  .391  **.023**  .227  .420 | 3.3 (1.7-6.4)  2.4 (1.0-5.6) | **.001**  **.044** |
| Time between admission to ICU and bacteremia, *days* | 11 [6-19] | 9 [5-15] | 1.0 (1.0-1.0) | .337 |  | |
| SAPS II admission score | 47 [32-57] | 48 [34-61] | 1.0 (1.0-1.0) | .558 |  | |
| SOFA score | 7 [5-11] | 13 [10-16] | 1.2 (1.1-1.3) | **<.001** | 1.1 (1.1-1.2) | **<.001** |
| Pitt bacteremia score | 6 [3-8] | 8 [7-9] | 1.2 (1.1-1.3) | **<.001** |  | |
| Septic shock | 12 (20) | 32 (65) | 4.1 (2.2-7.4) | **<.001** |  | |
| Mechanical ventilation | 40 (66) | 41 (84) | 2.1 (1.0-4.5) | .057 |  | |
| Duration of empirical therapy, *days* | 0 (0-2) | 1 (0-2) | 1.0 (0.9-1.2) | .719 |  | |
| Appropriate empirical therapy | 31 (58) | 28 (62) | 1.1 (0.6-2.0) | .785 |  | |
| Co-infections | 10 (17) | 11 (23) | 1.4 (0.7-2.7) | .349 |  | |
| Other antibiotic therapy | 11 (22) | 11 (25) | 1.3 (0.7-2.6) | .391 |  | |
| Time to effective antibiotic therapy, *hours* | 20 [5-44] | 16 [5-32] | 1.0 (1.0-1.0) | .706 |  | |
| Definitive antibiotic therapy with cefoxitin (versus carbapenem) | 36 (59) | 27 (55) | 0.9 (0.5-1.6) | .823 |  | |
| Data are presented as median [IQR] or count (%). P values in bold are statistically significant. Abbreviation as in Table 1. NA= not applicable  *Due to low patient numbers, infections related to liver abscess, skin and soft tissue, and neuromeningeal etiology were merged for the analysis. | | | | | | |


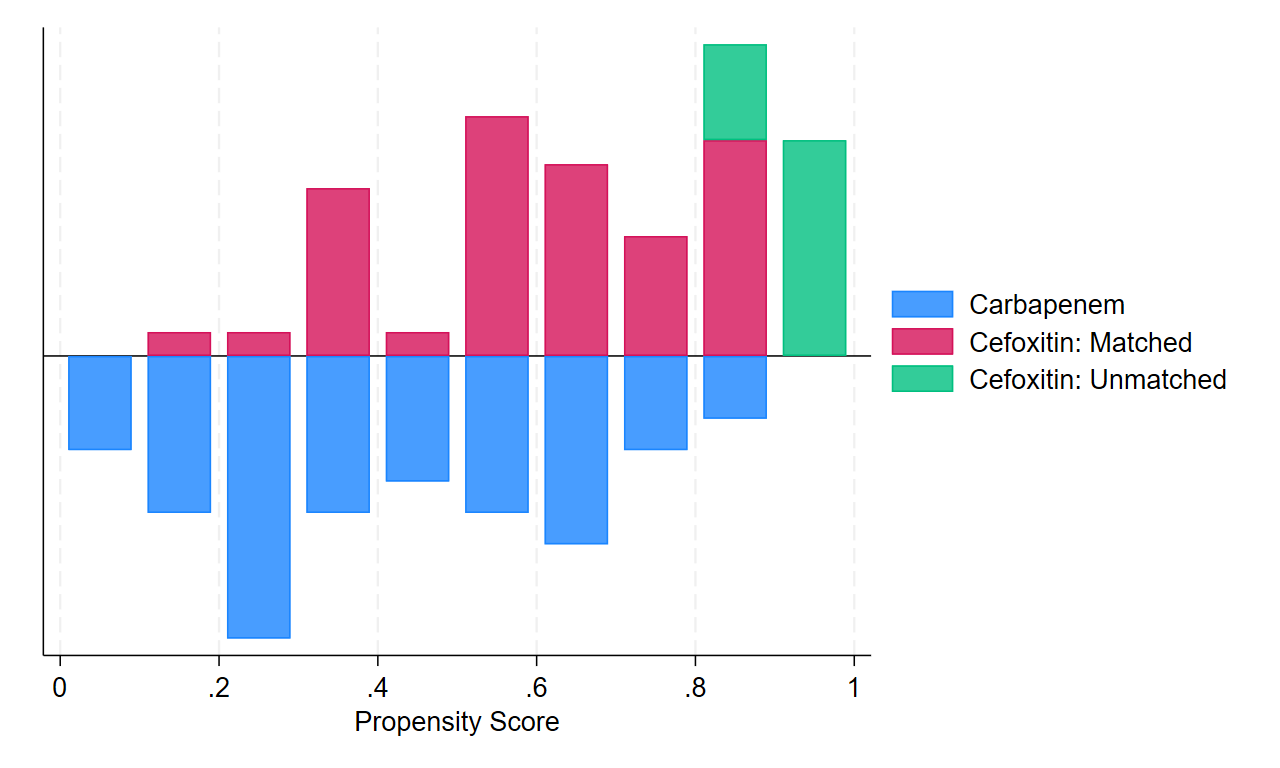


**Supplementary Figure 1 - Matching graph of the propensity score**
